# DNA Sequencing Methods: From Past to Present

**DOI:** 10.5152/eurasianjmed.2022.22280

**Published:** 2022-12-01

**Authors:** Kübra Eren, Nursema Taktakoğlu, Ibrahim Pirim

**Affiliations:** Faculty of Medicine, Department of Medical Biology and Genetic, Izmir Katip Celebi University, Izmir, Turkey

**Keywords:** DNA sequence,, Next-generation sequencing,, Illumina sequencing,, Ion torrent sequencing

## Abstract

Next-generation sequencing (NGS) is a highly effective genetic diagnostic test used in disease diagnosis. Although the Sanger method is used as the traditional method in genome studies, the use of NGS methods has been increasing with the development of technology. The foundation of next-generation sequencing was laid with the methods developed by Allan Maxam–Walter Gilbert and 2 Nobel laureates, Frederick Sanger. Initially, first-generation sequencing methods completed a certain part of the DNA with great efforts in a few days, while in today's technology, the entire DNA of even the most complex organisms is sequenced in 1 day. Second- and third-generation sequencing methods have been developed with improvements in cost, time, and accuracy of sequencing. The data obtained from these methods are interpreted with bioinformatics and contributed to the development of next-generation sequencing technology. These developments have increased the interest in studies on the relationship between next-generation sequencing and DNA or RNA depending on diseases. In this review, past and present methods of next-generation sequencing technologies are mentioned in detail and the difficulties and conveniences of these methods are reviewed.

**Main Points**

- Large-scale sequencing of genetic material of the human genome has been a subject of interest with the Human Genome Project. Therefore, next-generation sequencing technologies have been developed to analyze large amounts of data quickly, reliably, and cost-effectively.
- The basis of genome sequencing was established with the first-generation sequencing method. This generation is based on 2 methods: chain termination and chemical degradation.
- Roche 454, Illumina, SOLiD, and Ion Torrent platforms, which are second-generation sequencing techniques, were needed to reduce the cost and save time.
- Third-generation sequencing methods have emerged as technology has advanced, and the resulting data have been examined using bioinformatics to reduce the error rate.

## Introduction

DNA double helix structure was first explained by Watson and Crick. The first natural polynucleotide DNA sequence was announced in 1953. Due to the development of many sequencing techniques, the entire human genome was sequenced in 2003 as a result of the Human Genome Project.^1^ The goal of the Genome Project, a large-scale scientific initiative, is to investigate and evaluate the chemical sequence of the 50 000-100 000 genes that make up the human genome or the entire collection of all genetic material. The separation and examination of the genetic code included in DNA provide the basis for this huge project. Considering the size of the human genome, scientists must have developed new techniques for DNA analysis that can quickly, cheaply, and reliably analyze massive amounts of data. DNA sequencing methods requiring large-scale application have driven technology to both enhance capacity and decrease instrument size. This demand has prompted the creation of automated equipment that speeds up and lowers the cost of biochemical processes connected to sequencing, enhances the analysis of these reactions, and makes it simpler to enter the resulting data into databases.^2^ It was not possible for scientists at the time to sequence nearly a full gene because they could only sequence a few base pairs per year. Despite difficulties in the sequencing process, the first complete genome sequencing was completed with an explosion of RNA and DNA sequencing that improved procedures and provided new data. This eventually gave rise to the Maxam and Gilbert chemical degradation DNA sequencing method, which physically separates terminally tagged DNA fragments by electrophoresis and chemically cleaves specific bases of those pieces.^3^ Maxam-Gilbert, called the first-generation sequencing method, is based on chemical fragmentation of DNA and imaging with electrophoresis.^1^ Later, a new technique with higher simplicity, reliability, and a lower hazardous level was developed. It was simply called the Sanger sequencing method. Automated Sanger DNA sequencing with fluorescent dye labels predominated when the Human Genome Project was finished in 2003.^4^ Sanger technique, which uses fewer chemicals and is less harmful, is still regarded as the gold standard today.

Sequencing methods should be inexpensive, fast, accurate, and easy to implement. The inability of first-generation sequencing to meet these demands has led to new searches. The demand for less expensive and quicker sequencing techniques has grown since the first human genome sequence was completed. Massively parallel sequencing techniques avoid the scalability issues with standard Sanger sequencing by constructing micro-reactors and/or anchoring the DNA molecules to be sequenced to solid surfaces or beads. This enables millions of sequencing operations to occur simultaneously.^1^ After Sanger sequencing, next-generation sequencing (NGS) represents a real revolution in sequencing technology. A whole human genome may now be sequenced in a couple of days for less than $1000, compared to many years and billions of dollars for the first human genome's Sanger sequencing.^4^ Second-generation sequencing methods, also referred to as NGS, were developed in response to this demand. The quality of genome assemblies was greatly enhanced by the use of third-generation sequencers, which generate reads of previously unprecedented sequence lengths. Field sequencing is additionally made possible by quick sequencing and simple sample preparation.^5^ Millions of DNA fragments from a single sample are sequenced using the NGS technology known as massively parallel sequencing.^6^ To reduce the cost of sequencing and to enable the preparation, third-generation single-molecule sequencing has been created. The procedures involved in generating libraries, amplifying DNA, and sequencing that are part of the second-generation sequencing method are not necessary for the third-generation sequencing approach.

As a result, in this review, the evolution of DNA sequencing methods from the beginning to the present was discussed in detail. It was focused on how NGS methods referring to modern high-throughput sequencing processes sequencing methods actually developed and how they carried sequencing methods forward.

## First-Generation DNA Sequencing

First-generation DNA sequencing methods consist of 2 methods: Maxam-Gilbert (chemical degradation) and Sanger (chain termination) methods which are based on the amplification of template DNA and gel electrophoresis, emerged at approximately the same time, but the completion times differ from each other. These techniques that are crucial for sequencing the human genome currently have a disadvantage in terms of cost and time.

### Maxam–Gilbert Method

**Figure 1. A-C.**
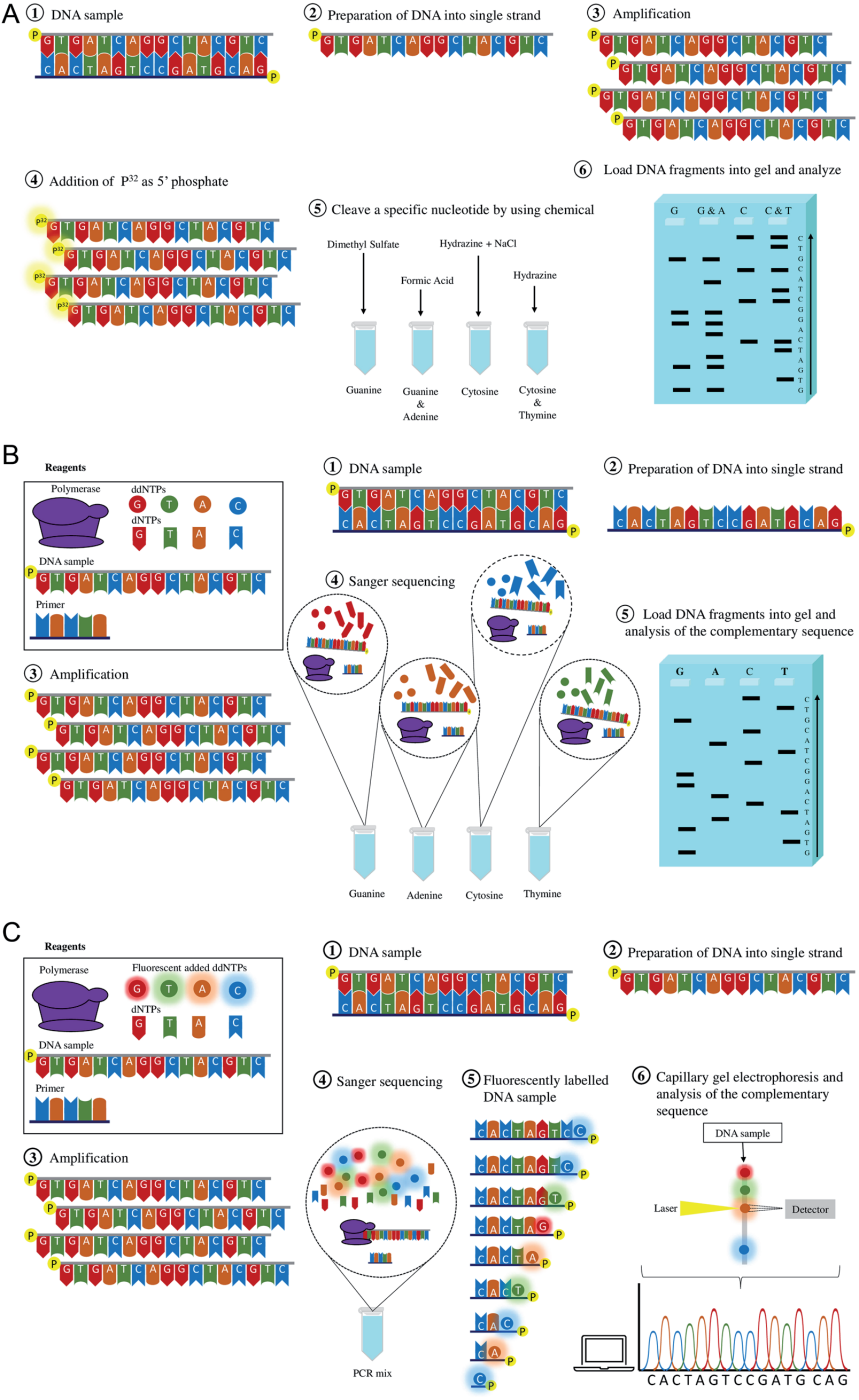
First-generation sequencing methods; (A) the basic principle of Maxam–Gilbert sequencing method, (B) schematic representation of the old Sanger method, (C) the new Sanger method in its simple form (G: guanine, A: adenine, C: cytosine, T: thymine, P: phosphate, P32: radioactive isotope of phosphorus, ddNTPs: dideoxynucleoside triphosphate, dNTPs: deoxyribonucleotide triphosphate, PCR: polymerase chain reaction).

The Maxam–Gilbert method is a method based on chemical degradation introduced by Maxam and Gilbert in the late 1970s. The first step is to convert the DNA sample into a single strand. The phosphate group in the DNA sequence is removed with the help of alkaline phosphatase.^7^ Polynucleotide kinase then adds a radioactive phosphate group (P^32^) to the single helical chain's 5' end.^8^ Millions of copies of the template DNA are produced by polymerase chain reaction (PCR). The replicated template DNA is cut into pieces by cutting on the base with base-specific chemicals.^9^ Four different chemicals are used for each base (A, G, T, and C). As a result, the reaction is conducted in 4 different tubes, 1 for each base. All tubes consist of base-specific chemical and radioactively labeled template DNA. The reading of the DNA sequences can be observed by placing the reactions on different strips of the gel with the help of electrophoresis. The negatively charged DNA samples are run on the gel with an electric current. To see the bands, the gel is placed on an x-ray film. The part with the radio-labeled sequences darkens on the x-ray film. The position of the bands that become visible will differ due to the chemical cut of the DNA samples. Since the sequence with a short base length will run fast, the sequence is read from the bottom to the up (Figure 1A).^9^

In the Maxam–Gilbert procedure, the guanine base is cut using dimethyl sulfate, the cytosine base is cut using hydrazine and sodium chloride, the guanine and adenine bases are cut together using formic acid, and the cytosine and thymine bases are cut together using hydrazine.^7,9,10^ The chemicals mentioned above methylate their specific bases. Only in high salt (NaCl) environment, hydrazine prevents methylation of the thymine base, thus making the distinction between thymine and cytosine bases easier. In addition, hot piperidine added to the medium breaks the chain of methylated DNA.^10^

The Maxam–Gilbert method is not routinely used in the laboratory due to various disadvantages^9^ such as high toxicity because of the phosphate isotope and cutting chemical usage, difficulties analyzing sequences longer than 500 bp, and errors during cleavage.^11^

### Sanger Method

The Sanger method, which was introduced by F. Sanger in 1977, is a method based on chain elongation termination, which is used in DNA sequencing with the help of polymerase and special nucleotides.^12,13^ The elongation of the chain is terminated by synthetic ddNTPs (dideoxynucleoside triphosphate; ddCTP, ddTTP, ddGTP, ddATP), which are the monomers of the DNA chain added to the reaction.^8,14,15^ The only difference between these ddNTPs and dNTPs (deoxynucleotide triphosphate; dCTP, dTTP, dGTP, dATP) is that they lack a hydroxyl group on the deoxyribose sugar's third carbon.^15^ Lack of hydroxyl causes polymerization to stop, and thus, chain elongation is stopped because it cannot establish a bond with the 5' phosphate of the following dNTP.^8^ Dideoxynucleoside triphosphates are designed for each nucleotide separately. Since each base corresponds to a different ddNTP reaction, the Sanger method of DNA sequencing creates a total of 4 distinct reactions for each base (Figure 1B). Template DNA, polymerase, primer, ddNTP, and dNTP are all included in each tube.^9,14^ Unlike dNTPs added to the reaction, the concentration of ddNTPs should be lower.^14^ When the polymerase adds 1 ddNTP to the elongated thread, no additional base can be added and the elongation stops.^9^ In this reaction carried out in parallel, loading is made into a separate well (4 different lanes) for each ddNTP on the polyacrylamide gel.^8^ In electrophoresis, the negative property of DNA is used and the positions of the DNA strands are determined.^9^ DNA strands transferred onto the gel form a ladder image when exposed to x-rays due to their different base lengths. Since the shortest thread will advance first, the reading is performed from the bottom up.^13^

With the advancement of technology, the Sanger method has begun to be performed with fluorescently labeled ddNTPs and capillary electrophoresis.^14^ While it was necessary to set up 4 different reactions for each base with the old method, the reaction is carried out only in a single tube with fluorescently labeled ddNTPs. Each ddNTP is labeled with 4 different base-specific fluorescent dyes.^13^ These fluorescently labeled ddNTPs provide information about the lastly added base to the DNA strand. By capillary gel electrophoresis, DNA fragments are sorted by size difference. The fluorescent dye at the terminator of ddNTPs on the last bases of the DNA sequences undergoing electrophoresis glows with a laser. Fluorescent detection occurs through spectral detectors called charge-coupled device (CCD).^15^ Software is required to interpret and analyze sequences (Figure 1C).^16^

The Sanger sequencing method is still valid today. According to the Maxam–Gilbert method, the use of hazardous chemicals and radioactive materials is limited. Therefore, it is a less toxic method. The automated Sanger method is the most commonly used one among DNA sequencing methods.^17^ The sequence synthesized in this method constitutes the complement of the target sequence.

## Second-Generation Sequencing

Second-generation sequencing technology allows for the rapid sequencing of whole genome. For this NGS, there are several different kits and equipment possibilities. These tools and kits are designed to make the procedures cost-effective and time-saving. As second-generation novel sequencing techniques, it makes use of the Roche 454, Illumina, SOLİD, and Ion Torrent platforms.

### Roche 454 Method

The Roche/454 GLS FLX Titanium instrument uses a platform known as pyrosequencing. Emulsion PCR and the subsequent pyrophosphate detection technique are the foundations of Roche/454 pyrosequencing.^18^ Nyrén et al^19^ introduced the pyrosequencing concept for the first time in 1997. It is a continuing procedure that combines magnetic beads covered with streptavidin, 3'-deficient recombinant DNA polymerase, and luciferase. 5'exonuclease activity (read-out evidence) and luminescence detection bring the process to a close.^20^

**Figure 2.**
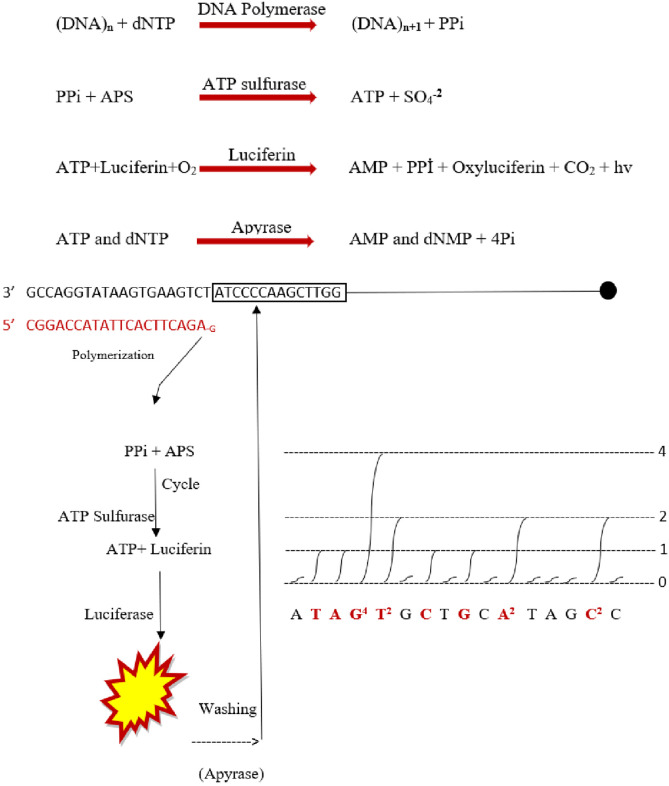
Principle of Roche 454 method (G: guanine, A: adenine, C: cytosine, T: thymine, dNTPs: deoxyribonucleotide triphosphate, ATP: adenosine triphosphate, AMP: adenosine monophosphate, dNMP: deoxyribonucleoside monophosphate, APS: adenosine 5′-phosphosulfate sodium salt, PPİ: pyrophosphate, Pi: phosphatase, hv: emission of light).

The 400-600 bp DNA fragments from the sequencing sample should be divided into libraries by adding adapter sequences to both ends.^21^ During the library-building process, PCR amplification by DNA polymerase lengthens each DNA fragment in the sample as nucleotides are added.^22^ In this method, each nucleotide must be added one by one to the reaction. Each amplification reaction occurs in a well containing a single DNA molecule in the form of a nanosphere or bead, oligonucleotides, or probes attached to its surface, a polymerase, and other essential elements (dNTPs, buffer, MgCl_2_, primers). The adapter sequences on the DNA fragments are complementary to the sequence of the probes. Only 1 DNA molecule can exist in each well for there to be a clonal amplification associated with each well. After library creation, the finished emulsion is placed in a picotiter plate (PTP). In each of the 100 000 wells that are individually observed during the pyrosequencing procedure, PTP only supplies 1 bead. Each well's data will be produced for a different DNA sequence. This procedure is called emulsion PCR (Figure 2).

### Illumina Method

Illumina acquired the Solexa Genome Analyzer and commercialized it in 2007, and today, the HiSeq and MiSeq platforms in particular are the most successful sequencing system on the market. The amplified template DNA for sequencing creates miniature colonies called polonies by bridging PCR.^16^

**Figure 3.**
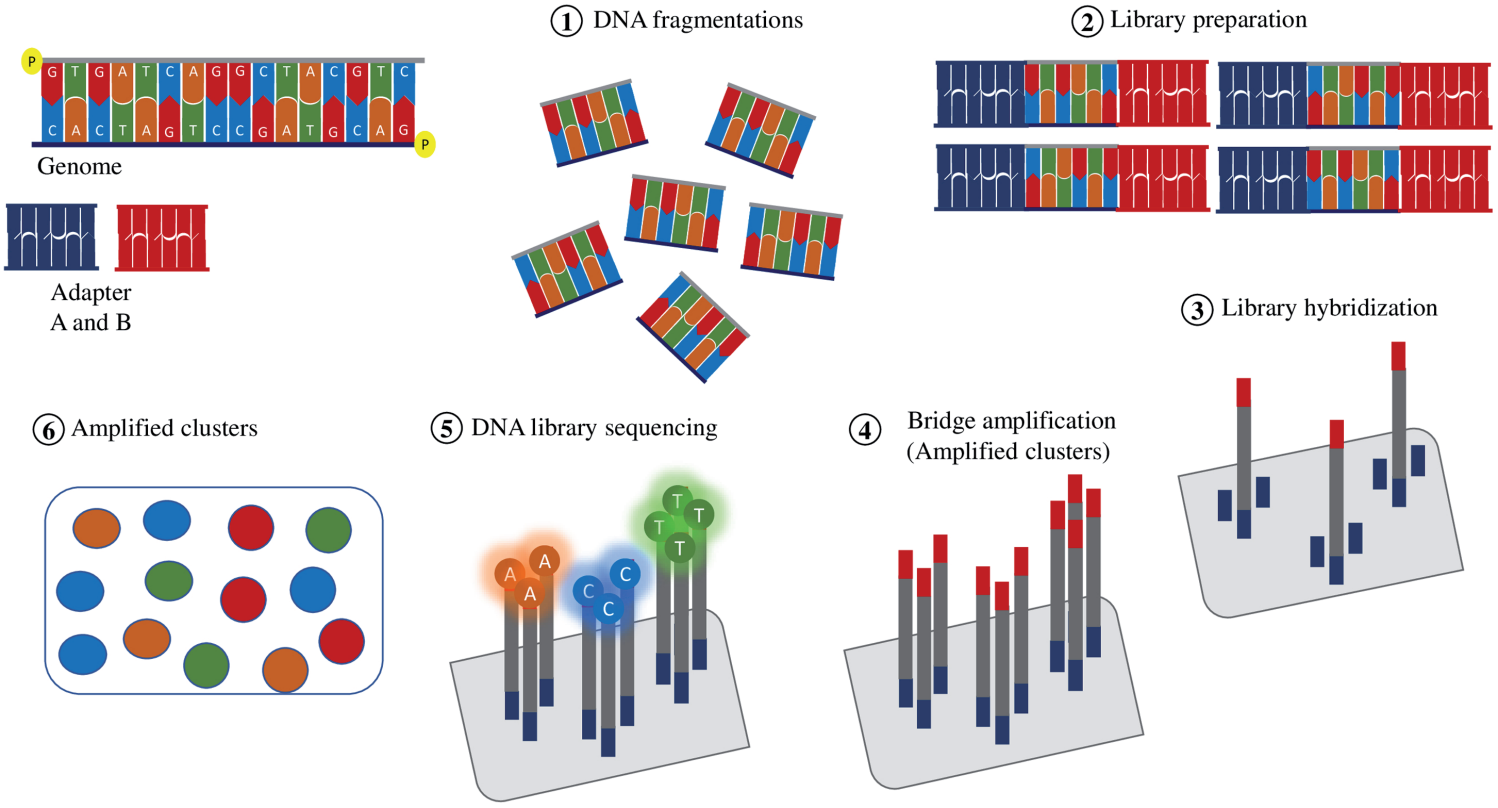
Library preparation and Illumina method (G: guanine, A: adenine, C: cytosine, T: thymine).

In order to sequence data, Illumina uses reversible dyes. When added to DNA strands, these colors make it possible to recognize nucleotides. The blocking group and fluorescence with a distinct color for each nucleotide are revealed when the nucleotide binds. To create DNA fragment clusters, the adaptor segments are joined to single strands and put in the flow cell. Bridge amplification is then carried out.^20^ The 4 types of nucleotides (A/T/C/G) are introduced, and each is fluorescently labeled with a different color. The unincorporated nucleotides are washed away as the 4 nucleotides compete for binding sites on the template DNA to be sequenced. Each synthesis is followed by the use of a laser to eliminate the fluorescent probe and blocking group. The subsequent cycle starts when 1 of the 4 bases develops a measurable fluorescent color distinct to it that allows for sequence identification. Up until the DNA molecule is sequenced, this process continues (Figure 3).

Scale differences between HiSeq and MiSeq technologies are substantial. In 10.8 days run, the HiSeq2000 produces more than 50 Gb of data and 1.6 billion base 100 dual-ended reads. MiSeq, in comparison, generates 1.5 Gb every day from 5 million 150-base paired-end reads and is intended for trials that last only 1 day.^16^

Compared to conventional sequencing techniques like Sanger sequencing, the Illumina approach has some advantages. Multiple sequences can be quickly sequenced at once using Illumina sequencing. Furthermore, unlike pyrosequencing, which requires expensive enzymes, this approach just employs DNA polymerase.^16^ With a 0.1% error rate, Illumina's reversible terminator technology and paired-end sequencing make it the most accurate base-by-base sequencing method available.^23^ Sequence data outperforms most other systems in terms of throughput, read length, cost, and run time.

### Solid Method

The ligation sequencing-based platform solid method was purchased by Applied Biosystems in 2006. A high rating of 99.85% indicates that the data collected through this platform's filtering is accurate.^20^

This technique also uses the Roche 454 emulsion PCR method, which uses tiny magnetic beads. When the DNA library is finished using PCR, the magnetic beads are affixed to a glass flow cell plate. The amplified fragments are sequenced using DNA ligase. These base pairs are found on two-base coded probes that have been specially created. These sensors are a mixture composed of an identifier base pair, a fluorescent molecule, and certain base components. Four distinct colors are defined to symbolize each base pair. The probe with the correct base pair is added to the sequence and bound to the primer by the ligase during the first cycle after the primer has been connected to the adapter. The fluorescent lights and the camera show the light as a result of binding. Following the removal of unbound probes, imaging of the fluctuating fluorescence that distinguishes the bound probe is completed. The sequential ligation cycle is restarted when the fluorescent dye has been removed.^21^

**Figure 4. A,B.**
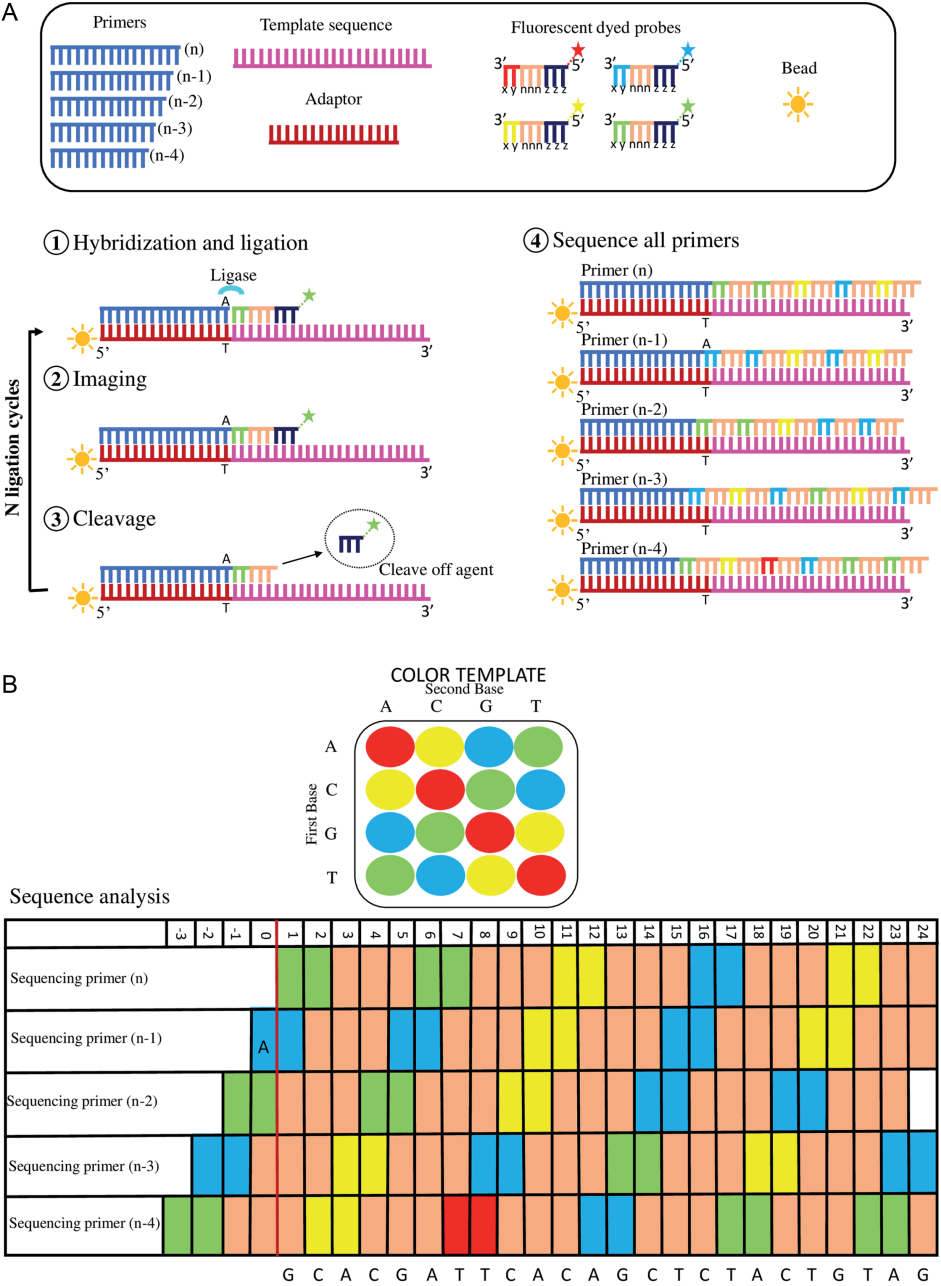
Solid Sequencing Method; (A) Stages of Solid method, (B) Analysis of DNA sequence with Solid method (G: guanine, A: adenine, C: cytosine, T: thymine, x: first base, y: second base, n: degenerate bases, z: universal bases, primer(n): GCGTAACGTAATGCTA).

The newly generated sequence, together with the primer, is entirely discarded, and the preceding procedures are started over with a new primer. New primer is attached to the main adapter that was previously handed to the medium one base back. The binding order in their detectors has been changed this time, which has resulted in various glow colors, because all of these other identifying base pairs bind one base back (n-1). During this time, the fluorescent color has been changing due to the new base pair. The nucleic acids determine according to this alteration. The known sequence is gradually completed with each cycle by shifting back the main base, and after 5 cycles, there is enough data to make up for any gaps in the sequence (Figure 4A).

Five different primers are used in the solid method. The base at the end of the primer is reduced by 1, completing the sequence in 5 cycles. The reason why there are 5 cycles is for the detection of degenerate bases in the prepared probes in other cycles. While designing the primer, the first base of the sequence is determined by the last base of the primer. It is known that the last base of the primer is adenine. Considering the color scale table, the second base is determined by looking at the base corresponding to the color of the first base (adenine). The first base of the other probe, which is in line with the second base, is determined. The same process continues for the others, and the DNA sequence is completed (Figure 4B). Solid sequencing method can be used in whole-genome sequencing, targeted region sequencing, and analysis of gene expressions and small RNAs.

Solid method can complete a single run in 7 days and generate 30 Gb of data in that time. Unfortunately, its main drawback is that read lengths are short and unsuitable for many applications.

### Ion Torrent Method

A primary base is shifted back in this method in each cycle, making the known sequence a little bit more complete at each step. After 5 cycles, there are enough information to fill in any gaps in the sequence. There are 5 ligation cycles in total. A reliable approach for sequencing can be applied to small RNA and gene expression analyses, whole-genome sequencing, and targeted region sequencing.^24^

This optics-based technique gathers photons from genome sequences and information on the appearance of the genome's base pairs. Conductive metals and software are combined in Ion Torrent (CMOS). Thus, the surface potential change of the metal oxide sensitive layer is stimulated and the potential of the terminal changes.^21^

**Figure 5.**
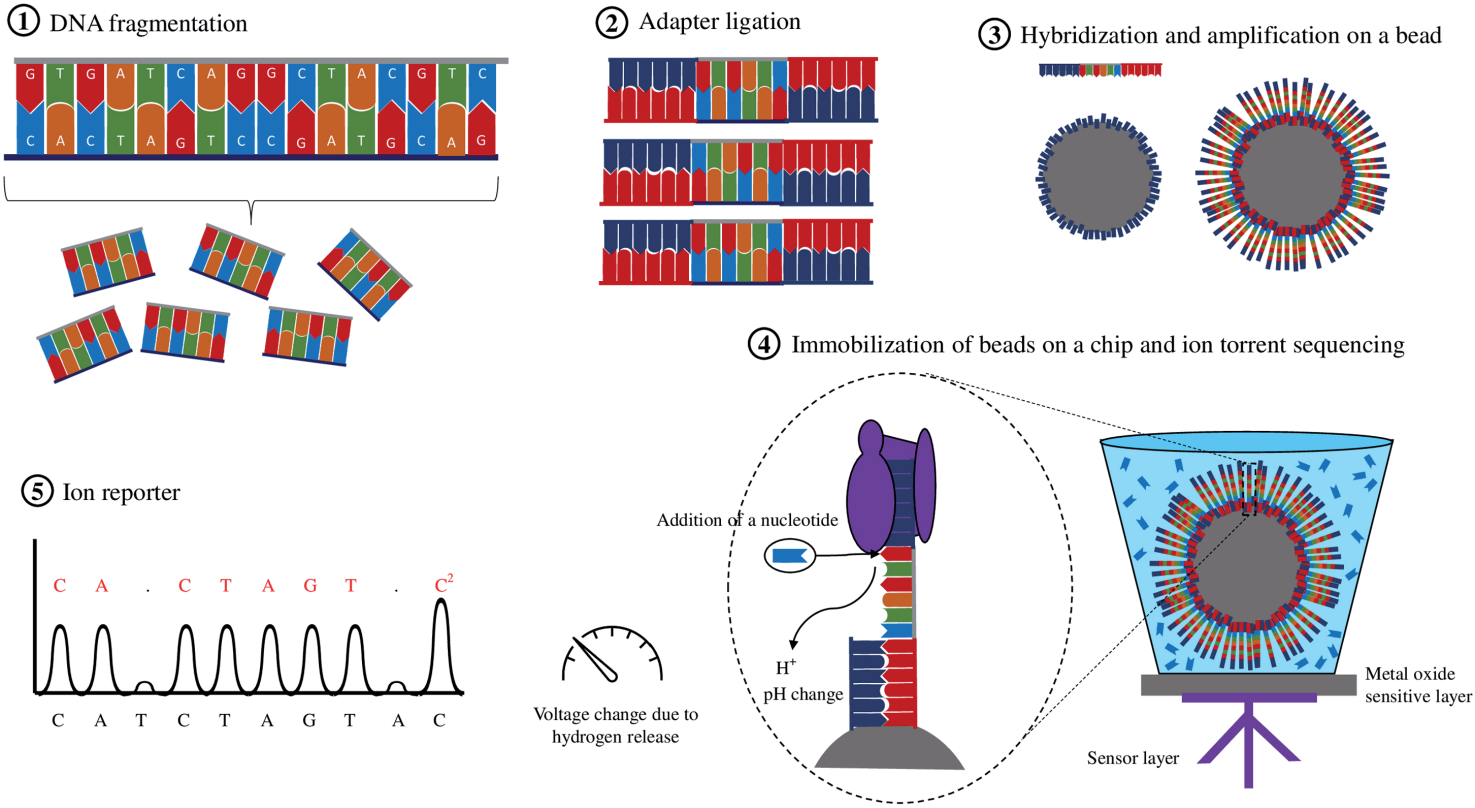
Ion Torrent sequencing and ion reporter (G: guanine, A: adenine, C: cytosine, T: thymine).

With the help of particular adapter sequences, fragmented DNA is joined to microbeads. Emulsion PCR beads are placed in a “chip” that has a million micro-wells, 1 bead in each one. A microchip with flow cells and electrical sensors below the cells hosts the sequencing reaction. Each nucleotide is incorporated and turned into an electrical signal. Protons are found using a semiconductor sensor that is ion-sensitive. A voltage signal proportional to pH is created from the sensor. Protons are found using a semiconductor and ion-sensitive semiconductor sensor. The sensor converts the pH fluctuations in the well into a voltage signal. Each well is integrated into the ion-sensitive layer of the chip and the ion sensor, allowing for the recording of very minute voltage changes caused by nucleotide addition during DNA synthesis sequencing. One by one, dNTPs are inserted into the wells. Unlabeled dNTPs are cleaned one after the other. Hydrogen ions are released as dNTP, which is complementary to the incoming nucleotide and is added to the chain. The pH of the solution varies as the H protons are released in direct proportion to the quantity of nucleotides (Figure 5).^21^

The system’s primary drawback is difficulty reading homopolymer extensions and repeats. Its main advantages seem to be relatively longer read lengths, more flexible workflow, more reduced time, and a more affordable price than those provided by other platforms.

## Third-Generation DNA Sequencing

With the increasing use of DNA sequencing methods, new technological developments are needed. Although first- and second-generation sequencing methods are revolutionary for DNA sequencing, they need to be improved in many aspects such as time, cost, and error rate. As a result of these searches, third-generation sequencing methods with longer read length, low cost and faster sequencing have been developed.^25,26^ The distinguishing features of the method are the fact that it doesn’t need amplification and allows real-time analysis without fragmenting the DNA (as a single molecule).^14,20,27^ Third-generation DNA sequencing methods are divided into two techniques as Pacific Bioscience and Oxford Nanopore Technology.

### Pacific Bioscience Method

**Figure 6. A,B.**
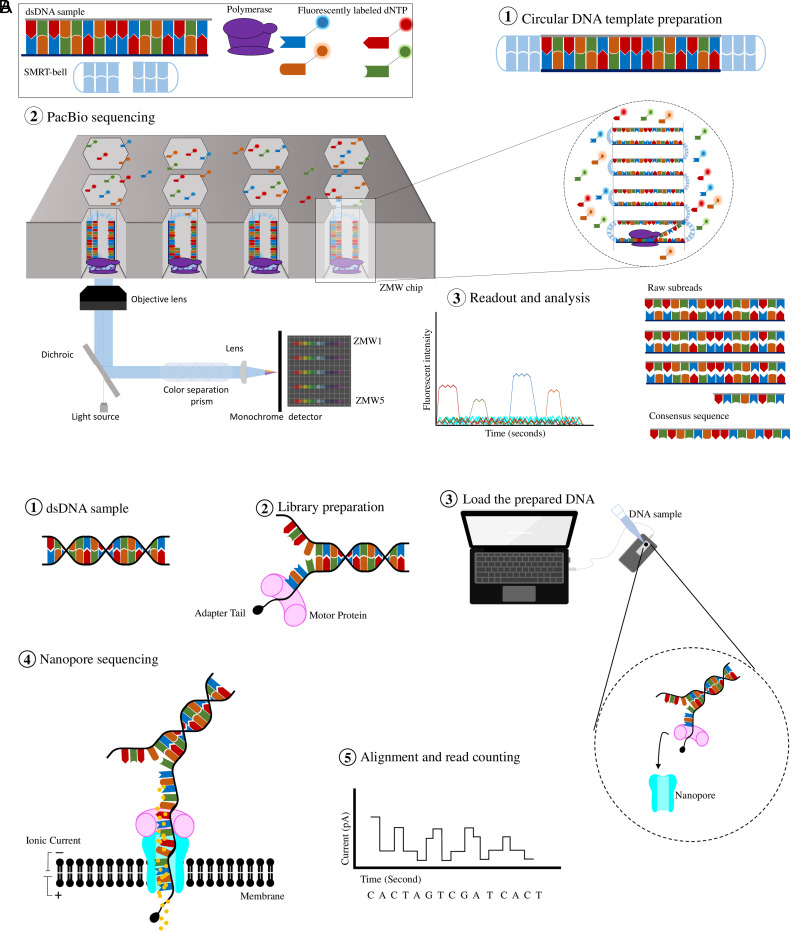
Third Generation Sequencing Methods; (A) the basic principle of PacBio sequencing method, (B) the Oxford nanopore technology method in its simple form (dsDNA: double-stranded DNA, SMRT: single molecule real-time, ZMW: zero mode waveguide, G: guanine, A: adenine, C: cytosine, T: thymine, pA: picoampere).

Pacific Biosciences' PacBio method, which was released at the end of 2010, is a single molecule real-time (SMRT)-based method.^14-16^ It is a method used to read long-dimensional DNA sequences by making use of the components necessary for DNA synthesis.^27^ In addition, the duration of preliminary preparation method is shorter than the other methods. The method involves inserting hairpin-shaped sequences known as “SMRTbell” at the beginning and end of a double-stranded DNA sample. Thanks to these sequences, the DNA becomes a circular sequence and minimizes base errors with long repetitive readings. The SMRTbell embedded DNA binds to polymerases that are immobilized to the base of a chip made of zero mode waveguides (ZMW) technology.^15,28^ In contrast to conventional DNA sequencing techniques, which sequence DNA by wrapping the polymerase around the DNA, this technique sequences DNA by immobilizing the polymerase to the well's bottom.^27^ There are thousands of picoliter ZMW wells on the chip and each well contains only 1 polymerase. Thus, a single DNA molecule is attached to only 1 well and can be analyzed in real-time. As the fluorescently labeled dNTP is incorporated into the DNA, a differently colored glow appears for each base.^15,20,28^ The signal ends with the cleavage of the nucleotide-bound fluorescent dye pyrophosphate by the polymerase.^25^ Imaging takes place instantaneously for each base added to the DNA.^16^ An optical system with green and red lasers at the bottom of the ZMW continuously observes the radiations in milliseconds. This process takes place in other wells simultaneously and in parallel. ZMW ensures that the light is distorted exponentially. As a result, fluorescent light only illuminates the well's bottom and does not diffuse into neighboring wells (Figure 6A).^15,16^

In addition to DNA sequencing, this method can detect epigenetic modifications and structural changes in the DNA sequence.^16,20^ Single molecule real time sequencing, which is still in development, is the most commonly used platform among third-generation sequencing methods.^8^ With the use of SMRTbell, the PacBio method showed its difference compared to other methods. While this method sorts quickly, it has a high error rate. The control of this problem is provided by reading the same sequence more than once and evaluating similar templates.^16^ The reading time varies depending on the length of the DNA but ranges from 0.5 to 4 hours.^25^

### Oxford Nanopore Technology Method

Another of the third-generation DNA sequencing methods is the Oxford nanopore technology (ONT) method developed by Oxford Nanopore Technologies. Despite the fact that this concept was first proposed in 1990, it has just recently been commercially available.^27^ This technique theoretically has a much lower mistake rate because it does not involve the use of enzymes, amplification, or fluorescently tagged nucleotides. For this method, an artificial nanopore embedded in the membrane with an electric current (100 pA-100 mV) is needed.^20^ This nanopore is formed by alpha-hemolysin (αHL), a 33 kD protein derived from *Staphylococcus aureus*.^17^ Before the DNA can be sequenced, the polyA tail-containing adapter sequence needs to be introduced into it. The adapter sequence is recognized and transported to the nanopore by a motor protein. Once the nanopore detects the adapter sequence, DNA begins to pass through the nanopore as a single strand. Since DNA is negatively charged, DNA moves through the nanopore with the ionic current passing through the membrane. Each of the bases that make up the genetic material will create a different interruption in the ionic current. The characterization of the molecule passing through the nanopore according to the interruption of the current is carried out by the algorithm (Figure 6B).^29^

The advantage of ONT over other methods is that it can read in a short time. In this method, analysis takes place without a secondary signal such as fluorescence, pH, or color. Since it does not need an enzyme like polymerase, it can sequence without exposure to high temperatures. In addition, since it does not require amplification, there is no long sample preparation step.^20^ In addition to being crucial for DNA sequencing, ONT is also crucial for characterizing RNAs, proteins, peptides, polymers, medicines, and macromolecules.^29^ The disadvantage of ONT is that the bioinformatics algorithm has difficulty processing the obtained data.^26^

## Future Approaches

Many complex diseases, including cancer, have been diagnosed and treated using sequencing techniques. Although methods such as reverse transcription PCR (RT-PCR), fluorescence in situ hybridization (FISH), and immunohistochemistry are used in the diagnosis of diseases, sequencing methods have a greater advantage. Sequencing methods can pave the way for new perspectives in disease diagnosis and treatment. Genetic variants that are currently unknown can be identified by sequencing. In order to find alternative treatment methods, changes in DNA, RNA, or protein levels can be examined by sequencing methods, mutations in cancer signaling pathways or repair mechanisms can be detected, and genetic profiles of individuals can be narrowed by comparing transcriptome readings of genetic materials obtained from patients and healthy individuals. As a result, the disease can be diagnosed early on, paving the way for personalized treatment methods.

Multiple sequencing methods have been introduced to date. These sequencing methods differ in terms of read speed, accuracy, and cost. Since DNA is a long material, the time, cost, and required storage space of the sequencing method increase proportionally.
